# Spatial Distribution Characteristics and Pollution Evaluation of Soil Iron in the Middle Hanjiang River

**DOI:** 10.3390/ijerph16214075

**Published:** 2019-10-23

**Authors:** Yuting Cheng, Peng Li, Guoce Xu, Kexin Lu, Feichao Wang, Tiegang Zhang, Zhaohong Feng

**Affiliations:** 1 State Key Laboratory of Eco-hydraulics in Northwest Arid Region, Xi’an University of Technology, Xi’an 710048, China; chengyutingstar@163.com (Y.C.); lipeng74@163.com (P.L.); lkx2942@163.com (K.L.); wangfeichao_0120@163.com (F.W.); fzhrose1012@163.com (Z.F.); 2 Key Laboratory of National Forestry Administration on Ecological Hydrology and Disaster Prevention in Arid Regions, Xi’an 710048, China; 3 Institute of Water Resources for Pastoral Area, Ministry of Water Resources, Huhhot 010020, China; zhang_tiegang@163.com

**Keywords:** soil iron, geostatistical, spatial interpolation, pollution assessment

## Abstract

Soil iron has an important impact on the ecological environment and on crop growth. This study selected a typical small watershed basin in the middle reaches of the Han River (Yujiehe) at Ankang City and used geostatistical methods and kriging interpolation to analyze the spatial distribution and structure of soil iron content for different land uses and at different depths, using the single-factor pollution evaluation to evaluate the pollution degree of soil iron. The results showed that soil iron in the Yujie River basin decreased with increasing soil depth, with contents of 8.80 mg/kg, 5.52 mg/kg, and 4.92 mg/kg at depths A1 (0–20 cm), A2 (20–40 cm), and A3 (40–60 cm). According to the classification index of effective trace elements in soil, the average contents of soil iron at these three depths were between 4.5 and 10 mg/kg, which are all considered moderate values. The coefficients of variation of soil iron at the three soil depths were 59%, 75%, and 83%, all of which showed moderate spatial variability, and the coefficient of variation increased gradually with soil depth. With semi-variance calculated at the three soil depths, soil iron optimal theoretical models were all exponential models with nugget coefficients of 9.52%, 47.76%, and 33.93%, indicating that spatial correlation was very strong in the A1 layer and moderate in the A2 and A3 layers. The spatial distribution of soil iron showed some variation in the study area, and the soil content was higher in the midwestern part in the A1 and A2 layers; however, in the A3 layer, the higher content was in the center and lower content was in the southern region. Correlations were significant between soil iron content on the one hand and land-use type and topographic factors on the other. The pollution indices of soil iron at the three soil depths under different land uses were all greater than 1.0, with the A1 layer in farmland being the worst, at 3.34. In the study area, using the background value of soil iron as an evaluation standard, the soil iron content of more than 65% of the Yujiehe region exceeded this standard.

## 1. Introduction

Fe is a necessary trace element for plant growth; it is an important component of various redox enzymes in plants and an essential element for synthesizing chlorophyll. When the Fe content in soil is too low, plant growth and development will be affected, leading to yellow-leaf disease in plants [1,2]. However, high Fe content can change the physiological state of plants and indirectly affect human health [3,4,5]. Soil is the main source of the requisite iron for plants, and under certain conditions, the iron content responds to the level of iron supply in the soil [6]. Although it is a small proportion of the trace elements in soil, it plays a very important role in soil fertility and environmental protection [7]; it also has a high degree of spatial variability, like other soil properties [8,9,10]. The soil total iron was generally not less than 1%, but this varies greatly depending on available iron in the soil; low content was 1–5 mg/kg and high content can be up to 1000 mg/kg [11]. Plants grown on calcareous soils are prone to iron deficiency chlorosis, which results in decreased crop yield and growth [12]. According to statistics, the area of soil with potential iron deficiency accounts for 25% to 30% of the world’s land area [13]. The effectiveness of iron mainly depends on the soil’s PH and redox potential. Large areas of calcareous soil are distributed in northwestern China. Due to the high PH and high bicarbonate concentration, the iron deficiency and chlorosis of plants are widespread [14]. At present, research on trace elements in soil has focused on overall elemental content [15,16] and studies of the available form are relatively few, especially for iron.

Despite the need for more research, the findings of this study suggest that the spatial variability of soil iron is mainly caused by random and systematic variation, indicating that this variability is related not only to natural factors, but also to human activities [17,18]. Soil iron content in farmland and irrigated fields is higher than in forested land, and soil pH, organic matter, and clay content have a great influence on the soil iron distribution [19,20]. Hence, the study of soil iron has mainly focused on different land uses, different functions and intensity, and different soil properties, but very few studies have analyzed its spatial distribution characteristics in combination with topographic factors in small watersheds, including pollution evaluation.

Soil iron is affected by plowing, the erosion–deposition–transportation process, soil properties, and soil element properties [21]. These factors cause a non-uniform spatial distribution of Fe, resulting in a deficiency or excess of iron in a particular area, with resulting impacts on plant growth. Information on the spatial distribution of soil total phosphorus and its relationship with land use and topographic factors at watershed scale is therefore of great significance to sustainable regional land use and the estimation of potential environmental pollution, as well as for protecting human health.

## 2. Description of the Study Area

**Figure 1:**
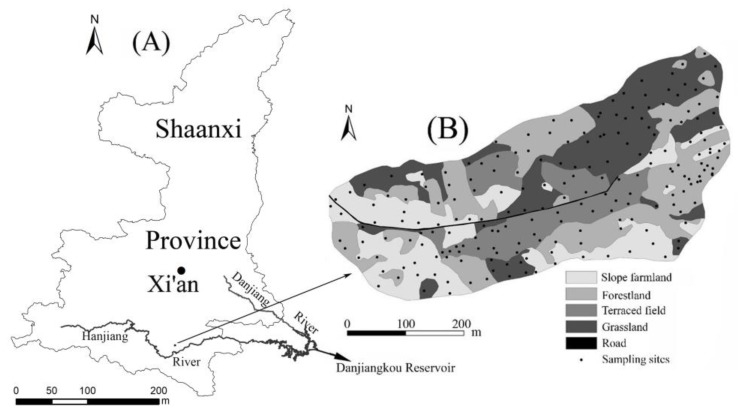
(**A**) Study area in Shaanxi province, China; (**B**) sampling sites and their land-use types.

The study was conducted in the Yujiehe watershed, which is part of the Han River basin, located in Ankang County in Shaanxi Province, China (108°48′15″–108°48′42″ E, 32°44′55″–32°45′13″ N). The area is 0.14 km^2^, and the elevation ranges from 310 m to 382 m, with a complex geological structure. The soil in the basin is mainly composed of weathered rock fragments mixed with sandy clay. Concentrations of nitrogen, phosphorus, and other nutrients are low, the soil is relatively poor, and the thickness of the soil layer is less than 70 cm. The main form of cultivated land is sloping farmland. The vegetation in the basin is mainly pine, oak, peach, and orange trees, and main crops include peanuts, corn, and peas. The average annual rainfall is 850 mm, but annual rainfall is unevenly distributed. The rainy season from July to October accounts for 65% of annual precipitation, whereas the precipitation in November to March accounts for less than 10%. The land-use type of sloping farmland accounts for 24% total land, 31% forest, 28% grassland, and 16% terraced fields. The distribution of land use and sampling points is shown in Figure 1.

In recent years, the government has carried out a soil and water conservation project in the Danjiangkou reservoir area. Since the Yujie River is highly representative in topography, vegetation, land use distribution, and industrial structure, the watershed has been selected as the project area. In the typical small watershed, slope engineering was carried out on changing mountain slope into terrace and steep slope farmland returning farmland to forest. The ecological environment has improved and has also promoted sustainable economic and social development in the basin. At present, the soil iron background value is 3.09 mg/kg in the basin, which is at a low level. Under the ecological construction conditions, whether the soil iron content has also changed, and whether there are obstacles to the current development of the agricultural economy requires further research.

## 3. Materials and Methods

### 3.1. Soil Sampling and Analysis

Soil samples were collected from 30 m × 30 m grids using a hand auger with a diameter of 5 cm in January 2014. A total of 205 samples were collected from depths of 0–60 cm and divided into three soil layers: A1 (0–20 cm), A2 (20–40 cm), and A3 (40–60 cm); 97 sampling points on the sunny slope and 108 sampling points on the shady slope. When the soil samples were collected, the land-use type, altitude, slope, and aspect were also recorded. The soil samples were taken back to the laboratory, and after natural air drying, they were ground and loaded into a plastic bag to be kept in reserve. Soil iron was determined using atomic absorption spectrometry.

In addition, 15 soil profiles were collected to a depth of 60 cm (A1 (0–20 cm), A2 (20–40 cm), and A3 (40–60 cm)). The soil profiles were all collected from grassland, forest land, and sloping farmland. The cores were weighed upon collection and weighed again after oven-drying at 105 °C for 24 h. Finally, the bulk density was calculated.

### 3.2. Data Analysis

Descriptive statistical analysis was performed using SPSS (20.0) (IBM Corporation, Armonk, New York, NY, USA), and the semi-variance function was calculated using GS+ (7.0) (Gamma Design, Plainwell, MI, USA.). Spatial distribution maps were created using ArcGIS (10.1) (Esri, Redlands, CA, USA).

The semi-variogram function is a key function to study soil spatial variability in geostatistics, and its theoretical model can be used to analyze the randomness and structure of spatial variation in soil iron [22]. Based on the regionalized variables theory and the intrinsic hypothesis, a semi-variogram, *γ*(*h*), can be expressed as follows:

(1) $$\gamma \left(h\right)=\frac{1}{2N\left(h\right)}{\sum_{i=1}^{N\left(h\right)}\left[z\left(x_{i}\right)-z\left(x_{i}+h\right)\right]}^{2}$$

where *N(h)* is the number of pairs of observations [*z*(*x_i_*); *z*(*x_i_* + *h*)] separated by distance *h*. A second software package, GS+ (Gamma Design, Plainwell, MI, USA), was then used to measure geostatistical parameters at distances closer than the sampling interval (30 m in the present study), including C_0_: structural variance, C_1_: sill variance (C_0_ + C_1_), and spatial autocorrelation length (range), which were derived from the fitted semi-variograms. The degree of spatial dependence (GD) can be used to divide spatial dependence into distinct classes: if GD is less than 25%, the variable is considered strongly spatially dependent; if between 25% and 75%, moderately so; and if more than 75%, weakly spatially dependent. The degree of spatial dependence was calculated as follows:

(2) $${\mathrm{GD}=C}_{0}{/(C}_{0}{+C}_{1})\times 100\%$$

A single-factor pollution evaluation method was used to evaluate soil iron pollution [23]. The calculation formula was:

(3) $$P_{i}=\frac{C_{i}}{S_{i}}$$

where *P*_i_ is the pollution index of iron, *C*_i_ is the measured concentration of iron, and *S*_i_ is the evaluation criterion for iron. The background value of local elements is generally assumed. The background value used in this study was 3.09 mg/kg.

## 4. Results and Discussion

### 4.1. Descriptive Statistics of Soil Iron

Descriptive statistics of soil iron content at different soil depths are listed in Table 1. The mean soil iron content decreased with increasing soil depth, and there were significant differences among the A1, A2, and A3 soil layers (*p* < 0.01), where the contents were 8.80 mg/kg, 5.52 mg/kg, and 4.92 mg/kg, respectively. This showed that soil iron was enriched at the soil surface. Since the soil surface contained a large amount of dead leaves and crop straw mulch, it could ensure a warm and moist soil environment, which was conducive to the growth of microorganisms and the formation of soil organic matter. Organic matter is an iron-complexing agent, and complexing iron is an important source of soil iron [5]. Soil organic matter is also the main carrier of adsorption cations, which can lead to adsorption of soil iron [24]. In addition, with increasing soil depth, the number of plant roots gradually increases, and roots consume large amounts of soil iron [25]. Therefore, in the A2 and A3 soil layers, iron content was less than in A1. According to the grading standard of soil effective microelements in China, the iron content can be divided into five grades: extremely low (<2.5 mg/kg), low (2.5–4.5 mg/kg), moderate (4.5–10 mg/kg), rich (10–20 mg/kg), and extremely rich (>20 mg/kg) [26,27]. The average content of soil iron at the three soil depths was between 4.5 and 10 mg/kg, which is at the moderate level.

The coefficients of variation (CV) for the A1, A2, and A3 soil layers were 59%, 75%, and 59%, respectively. According to Nielsen and Bouma [28], the variability of soil iron in the three soil layers is moderate and increases with increasing depth. This indicates that the spatial variation of soil iron is moderate and that the degree of dispersion increased with soil depth; human disturbance also gradually increases soil iron [20].

**Figure 2:**
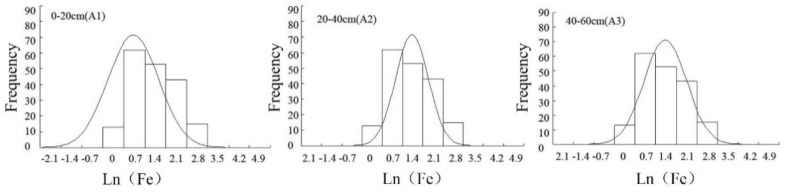
Histograms of soil iron at different soil depths.

Data must be tested for normality before geo-statistical analysis. According to the kurtosis and skewness coefficients and the K-S (Kolmogorov-Smirnov) test reported in Table 1, soil iron does not satisfy a normal distribution for the three soil layers [29]. This was the case because in the process of soil formation, due to the influence of external factors, the soil characteristics changed and soil iron moved and redistributed, changing the original uniform distribution. Therefore, the data was subjected to a logarithmic transformation. After transformation, the *p*-values of the K-S test were 0.082, 0.117, and 0.141, showing that the data at the three depths were normally distributed (*p* > 0.05). Histograms of soil iron at different soil depths are shown in Figure 2.

### 4.2. Semi-Variogram Analyses of Soil Iron

**Figure 3:**
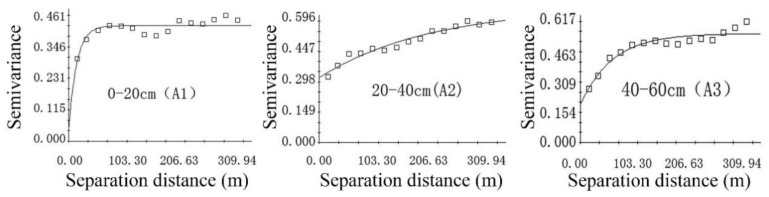
Semi-variogram function theoretical model of soil iron at different depths.

Descriptive statistical analysis of soil iron can only show the change characteristics of iron content; it cannot reflect spatial, structural, and correlation characteristics. Therefore, the spatial variation of iron was analyzed by geo-statistical means. After data transformation, the iron obeyed a normal distribution and satisfied the requirements of semi-variance function analysis. The exponential model with the highest degree of fit (R^2^) and the smallest residual sum of squares (RSS) was selected as the optimal theoretical model [22]. The relevant parameter values of the model are shown in Table 2 and Figure 3.

The results showed that the nugget coefficients of soil iron were 9.52%, 47.76%, and 33.93%, respectively at the depths of the three soil layers. The nugget coefficient was the smallest in A1, indicating that the distribution of iron was mainly controlled by internal structural factors such as soil properties, climate, and topography; these natural factors can lead to strong spatial correlations of soil nutrients. The spatial correlations in A2 and A3 were moderate, indicating that the iron in layers A2 and A3 was affected not only by structural factors, but also by random factors such as different farming measures, planting systems, fertilization, and other human activity [30].

### 4.3. Spatial Distribution of Soil Iron

**Figure 4:**
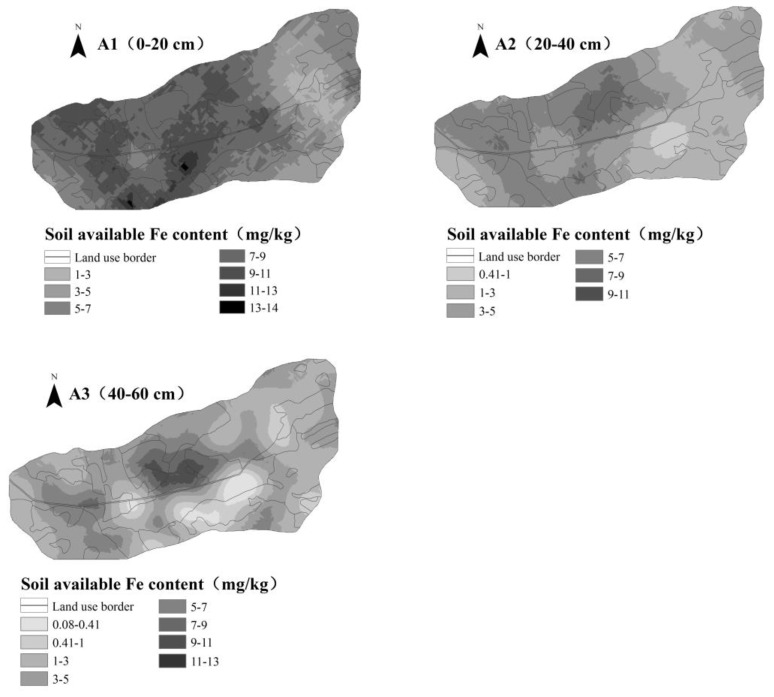
Spatial distribution of soil iron content at different soil depths.

According to the semi-variogram function model of soil iron, the kriging interpolation method was used to interpolate iron optimally over the three soil depths. The spatial distribution of soil iron at different soil depths is plotted in Figure 4. It is clear that the highest values of soil iron in the A1 layer were mainly found in a small area of the southern region of the basin. Generally speaking, the soil iron content in the central and western regions was higher than in the eastern region because the central and western regions are mainly agricultural land, whereas close to residential areas, the discharge of domestic garbage has a certain impact on soil iron. On the other hand, the soil iron content in the eastern part is lower because the eastern part is mainly forest land and grassland; the vegetation pattern was relatively simple and was not conducive to the formation of soil organic matter.

In general, soil iron in the A2 layer was higher in the central and western regions than in the eastern region. The highest values of soil iron were mainly distributed in the north-central region. This area was mainly terraced, and soil erosion was minimal. Therefore, soil iron was not easily lost with water and soil. At the same time, it was also closely related to soil texture. Soil organic matter content was high in this area, and large iron ions were easily adsorbed [31]. The soil iron distribution in the A3 layer was different from that in A1 and A2 layers, taking the shape of an island. This was because under the influence of factors such as human cultivation and fertilization activities, soil iron gradually deposits along with the depth of the soil layer. At the same time, due to the physiological activities of rhizosphere microorganisms and the action of root exudates, a large amount of organic matter exists in the rhizosphere soil which increases the solubility of insoluble iron in the soil [32]. The highest values of soil iron were mainly distributed in the center of the basin, whereas the south-central region had the lowest values. This was the case because the central area of the basin has the lowest altitude, to which surface runoff was directed. Soil moisture content increased, causing soil iron to reduce to dissolved ferrous ions, thereby increasing soil iron content [32].

### 4.4. Soil Iron and Bulk Density under Different Land-Use Types

When the land-use pattern changes, soil iron content and its dynamics also change. The soil iron content and bulk density under different land uses are shown in Table 3. Clearly, soil iron content conformed to the following order in the A1 layer: farmland > forest land > grassland; whereas in the A2 and A3 layers, it conformed to the following order: farmland > grassland > forest land. This pattern occurred because farmland underwent more fertilization, artificial cultivation was frequent, and the large amount of animal, plant, and crop straw residues in the soil provided a good living environment for microorganisms, contributing to the formation of soil organic matter and increasing soil iron content [33]. However, the vegetation patterns of forest and grassland were relatively simple, soil clay content was less, organic matter content was low, and plant root systems (which consume more iron) were more developed, making the iron content lower [32].

The ANOVA test found an extremely significant difference (*p* < 0.01) in soil iron under different land uses in A1 (*p* < 0.01). However, there was no significant difference in soil iron content under different land uses in the A2 and A3 layers (*p* > 0.05), but there were significant differences in soil iron content between the different soil layers (*p* < 0.01). This indicated that land use had a significant effect on the content of iron in surface soil but had no obvious effect on soil iron content in the A2 and A3 layers. Under the same land use, the correlations between iron content in each soil depth were strong, and the soil layers influenced each other. The soil bulk density under different land uses showed the order: grassland > farmland > forest land in the A1 and A2 layers but showed the order: grassland = forest land > farmland in the A3 layer. With increasing soil depth, the soil bulk density gradually increased.

### 4.5. Correlation between Soil Iron and Topographic Factors

Topographic factors have a certain effect on soil iron content. The relationship between soil iron content and altitude, slope, and aspect under different soil depths is shown in Table 4. Clearly, slope, aspect, and elevation have different effects on soil iron. There was no correlation between iron content and aspect in the A1, A2, or A3 layers. This was the case because humans’ different farming practices, planting patterns, and fertilization had weakened the effect of slope direction on soil iron content.

However, the iron content at different soil depths showed a significant negative correlation with altitude because as altitude increases, the temperature gradually decreases, and the metabolism of animals and plants decreases accordingly, leading to a decrease in soil organic matter and also in soil iron. Accordingly, weeds cover the high elevation in the study area and human activities are less. In addition, under natural conditions, soil iron migrates to the lower elevation with the rainfall runoff erosion and is enriched at lower altitudes. Thus, soil iron content is reduced as the altitude increases [32]. Soil iron was significantly correlated with slope (*p* < 0.01) in the A1 and A2 layers, but not in the A3 layer. This pattern occurred because vegetation coverage decreased with increasing slope in the study area, and the loss of surface water, soil, and nutrients on steep slopes was serious. The A3 layer was mainly affected by plant roots and rainfall leaching, eliminating the influence of slope, and therefore its correlation was not significant.

### 4.6. Evaluation of Soil Iron Pollution

The evaluation results for the soil iron pollution index are shown in Table 5. The background value of iron was 3.09 mg/kg in the study area, and the percentages by which the A1, A2, and A3 soil layers exceeded this background value were 87%, 63%, and 57%, respectively. As soil depth increased, the percentage above the background value decreased. The soil iron pollution index was greater than one for all three soil layers under different land uses in the Yujie River basin. The pollution of farmland was serious (3.3 times higher than the background value), followed by forest land and grassland, which were 2.8 times and 2.3 times higher respectively than the background value. Therefore, the soil iron content exceeded the standard in more than 65% of the study area, meaning that reasonable measures should be taken to control soil iron pollution to ensure soil quality.

## 5. Conclusions

Soil iron content decreased with increasing soil depth in the Yujie River basin. The spatial variation of soil iron was moderate for all three soil depths, and the coefficient of variation gradually increased for deeper soil layers. Semi-variogram analysis showed that for predicting soil iron, the exponential model was the best for layers A1, A2, and A3. In the layer A1, soil iron was strongly space-dependent, the highest soil iron being distributed mainly in the southern part of the basin. Land use had a great impact on the distribution of soil iron: it was the highest under farmland, followed by forest land, and grassland; soil iron was also significantly higher, as shown by the *t*-test (*p* < 0.05), in layer A1 than that in layers A2 and A3. Soil iron was significantly and negatively correlated to altitude and positively correlated to slope. Soil iron pollution was serious in farmland, followed by forest land, and grassland, and in the study area soil iron content exceeded the standard in more than 65%. This result can provide a method reference and theoretical basis for predicting the soil iron content spatial distribution in the rocky mountain area.

## Figures and Tables

**Table 1:** Descriptive statistics of soil iron content under different soil depths (mg·kg^−1^).

Soil Layer	Mean	SD	Skewness/Kurtosis	Minimum	Maximum	K-S (*p*)	CV (%)
A1	8.80	5.16	0.42/−1.03	1.60	20.59	0.014	59
A2	5.52	4.14	1.16/0.81	0	20.73	0.00	75
A3	4.92	4.11	1.51/2.19	0	21.34	0.00	83

Note: SD: Standard Deviation; K-S: Kolmogorov-Smirnov test; CV: coefficient of variation.

**Table 2:** Geostatistical parameters of soil iron at different depths.

Soil Layer (cm)	Nugget Value	Base Station Value	Nugget Coefficient (%)	Range (m)	Model	R^2^	RSS
A1	0.04	0.42	9.52	54	Exponential	0.715	6.101 × 10^−3^
A2	0.32	0.67	47.76	193	Exponential	0.957	3.924 × 10^−3^
A3	0.19	0.56	33.93	58	Exponential	0.911	9.748 × 10^−3^

Note: RSS, the smallest residual sum of squares.

**Table 3:** Average soil iron content and bulk density of soil under different land uses.

Soil Layer	Grassland		Farmland		Forest Land	
	Soil Iron (mg/kg)	Bulk Density (g/cm^3^)	Soil Iron (mg/kg)	Bulk Density (g/cm^3^)	Soil Iron (mg/kg)	Bulk Density (g/cm^3^)
A1	7.21	1.44	10.33	1.31	8.78	1.29
A2	5.46	1.56	6.11	1.55	5.26	1.53
A3	5.33	1.61	5.73	1.59	4.33	1.61

**Table 4:** Correlation analysis of soil iron contents with different topographic factors.

Soil Layer	Altitude	Slope	Aspect
A1	−0.229 **	0.263 **	−0.029
A2	−0.279 **	0.240 **	−0.047
A3	−0.152 *	0.120	0.044

Note: * Indicates significant correlation, *p* < 0.05; ** indicates extremely significant correlation, *p* < 0.01.

**Table 5:** Soil iron content and pollution index under different land uses.

Soil Layer	Grassland		Farmland		Forest Land	
	Soil Iron (mg/kg)	Pollution Index	Soil Iron (mg/kg)	Pollution Index	Soil Iron (mg/kg)	Pollution Index
A1	7.21	2.33	10.33	3.34	8.78	2.84
A2	5.46	1.77	6.11	1.98	5.26	1.70
A3	5.33	1.72	5.73	1.85	4.33	1.40
